# PM Source Apportionment for Short-Term Cardiac Function Changes in ApoE^−/−^ Mice

**DOI:** 10.1289/ehp.8091

**Published:** 2005-07-05

**Authors:** Morton Lippmann, Jiang-Shiang Hwang, Polina Maciejczyk, Lung-Chi Chen

**Affiliations:** 1 New York University School of Medicine, Tuxedo, New York, USA; 2 Institute of Statistical Science, Academia Sinica, Taipei, Taiwan

**Keywords:** concentrated ambient particulate matter, heart rate, heart rate variability, motor vehicle pollution, PM_2.5_, residual oil, resuspended soil, secondary sulfate, source apportionment

## Abstract

Daily rates of cardiovascular mortality and morbidity are have been associated with daily variations in fine particulate matter (aerodynamic diameter ≤2.5 μm, PM_2.5_), but little is known about the influences of the individual source-related PM_2.5_ categories or the temporal lags for the effects. We investigated heart rate (HR) and HR variability (HRV) data collected during a 5-month study involving 6 hr/day, 5 day/week exposures of normal (C57) mice and a murine model for atherosclerotic disease (ApoE^−/−^) in Sterling Forest (Tuxedo, New York, USA). The mice were exposed to concentrated ambient particles (PM_2.5_ concentrated 10-fold, producing an average of 113 μg/m3). Daily 6-hr PM_2.5_ air samples were analyzed by X-ray fluorescence, permitting attribution to major PM source categories [secondary sulfate (SS), resuspended soil (RS), residual oil (RO) combustion, and other, largely due to motor vehicle traffic]. We examined associations between these PM_2.5_ components and both HR and HRV for three different daily time periods: during exposure, the afternoon after exposure, and late at night. For HR there were significant transient associations for RS during exposure, and for SS in the afternoon after exposure. For HRV, there were comparable associations with RO in the afternoon after exposure and for both SS and RS late at night. The biologic bases for these associations and their temporal lags are not known but may be related to the differential solubility of the biologically active PM components at the respiratory epithelia and their access to cells that release mediators that reach the cardiovascular system. Clearly, further research to elucidate the underlying processes is needed.

Many published studies have demonstrated statistically significant associations between ambient air fine particulate matter (aerodynamic diameter ≤2.5 μm, PM_2.5_) mass concentrations and short-term changes in heart rate (HR) and/or HR variability (HRV) in humans and laboratory animals (U.S. Environmental Protection Agency 2004). However, interpretation of these findings is complicated in that the effects may go in either direction and are observed on some days and not on others. These inconsistencies may be due to the limitations of PM_2.5_ mass as an index of exposure to the biologically active components of the ambient PM_2.5_. It is well known that the composition of ambient air PM_2.5_ has considerable temporal and spatial variability. Studies in human volunteers and laboratory animals have been limited in their power to identify the causal components because they require both the long-term collection of electrocardiographic (ECG) data and simultaneous availability of data on tracers or factors associated with PM_2.5_ composition.

In a recent study of the effects of sub-chronic (5–6 months of daily 6 hr) exposures of normal (C57) mice and a mouse model of atherosclerosis (apolipoprotein deficient, ApoE^−/−^, mice) to fine concentrated ambient particles (CAPs) in Tuxedo, New York, USA, at an average concentration of 113 μg/m^3^, we generated the kinds and amounts of data needed to address the issue raised in this article. The results of the overall subchronic study design and the results obtained for associations of PM_2.5_ mass concentration with progressive changes in HR and HRV and the changes in atherosclerotic plaque, gene expression, and brain cell distribution at the end of the study are described in a series of articles (Chen and Hwang 2005; Chen and Nadziejko 2005; Gunnison and Chen 2005; Hwang et al. 2005; Lippmann et al. 2005a 2005b; Maciejczyk et al. 2005; Veronesi et al. 2005). In another article (Maciejczyk and Chen 2005) describing the parallel study that went on simultaneously with the inhalation study, we exposed BEAS-2B cells (an airway epithelial cell line) *in vitro* to CAPs and reported that nuclear factor-kappa B (NFκB) expression was most closely associated with the residual oil (RO) component, which was, on average, 1.4% of the PM_2.5_ mass.

For this article, we used the 5 months of daily 6-hr source apportionments described in Maciejczyk and Chen (2005), the continuous HR data for exposure (weekday) days provided in Hwang et al. (2005), and the corresponding HRV data given in Chen and Hwang (2005) to determine the source-related PM_2.5_ components’ short-term associations with HR and HRV.

## Materials and Methods

The methods used to generate the factors associated with specific major PM source categories were described by Maciejczyk and Chen (2005). Briefly, fine CAPs were collected from a rural area upwind of New York City for the 0900- to 1500-hr period on weekdays only, March through September 2003. Chemical composition data for CAPs were modeled using factor analysis with varimax orthogonal rotation to determine four particle source categories contributing significant amount of mass to CAPs at Sterling Forest (Tuxedo, New York). These source categories are regional secondary sulfate (SS) characterized by high sulfur, silicon, and organic carbon; resuspended soil (RS) characterized by high concentrations of calcium, iron, aluminum, and silicon; RO-fired power plants emissions of the Eastern United States identified by presence of vanadium, nickel, and selenium; and motor vehicle (MV) traffic and unknown other sources. To estimate the mass contributions of each individual source category, the CAP mass concentration was regressed against the factor scores. Using the method developed by Thurston and Spengler (1985), we determined that regional sulfate was the largest contributor to average mass (56.1%), followed by soil (11.7%). The RO combustion accounted for 1.4%, and the MV traffic and other sources category contributed 30.9%.

The methods used to process the voluminous HR and HRV data for the same period were described by Hwang et al. (2005) and Chen and Hwang (2005). Briefly, they used their recently developed nonparametric method (Nadziejko et al. 2004) to estimate the daily time periods that mean HR differed significantly between the CAPs and the air sham-exposed groups. CAP exposure most affected HR between 0130 and 0430 hr. With the response variables being the average HR, they adopted a two-stage modeling approach to obtain the estimates of chronic and acute effects on the changes of this variable. In the first stage, a time-varying model estimated daily crude effects. In the second stage the true mean of the estimated crude effects was modeled with a polynomial function of time for chronic effects, a linear term of daily CAP exposure concentrations for acute effects, and a random component for unknown noise. A Bayesian framework combined these two stages.

For the analyses of HRV, the times in milliseconds of occurrence of two consecutive R waves in the ECG waveform (RR) were calculated on a beat-to-beat basis. Because of limitation in data storage capacity, the RR intervals were recorded consecutively for 5 sec in every 15-min interval for all mice during 10–27 April 2003, and for ApoE^−/−^ mice in the control group during 22 April through 20 July 2003. The rest of recordings were taken consecutively about 10 sec in every 5-min interval for the mice. There are about 34–64 and 100 RR intervals recorded in 15-and 5-min intervals, respectively. For the analysis, we decided to work on fluctuations of RR intervals on an every 15-min basis. To match the data in the 15-min recordings, we used only the first 60 RR intervals in the last of 3 consecutive 5-min intervals. The two HRV indices that we used were the standard deviations of the RR intervals (SDNNs) and the square root of the mean squared differences (RMSSD) of successive RR intervals in 5 sec. The nonparametric method identified the 0000- to 0500-hr period during which the two groups had the largest HRV differences within each day. To match the HR analyses of effects with the HRV changes, we used the same period (0130–0430 hr) for calculating mean log SDNN and log RMSSD to represent daily HRV responses for this period for each mouse. In the analysis of effects on HR, we also calculated daily responses for the 1100- to 1300-hr period during exposure for examining acute effects. However, because the number of normal RR intervals recorded during the exposure period was small because of interference from the perforated metal chamber, we instead used the 1600- to 1800-hr interval after exposure as an alternate for calculating daily HRV response. Daily changes in HR during this period, which were not reported in the previous study, were also calculated for this analysis.

To examine whether variations of concentrations in major sources are correlated with short-term changes of cardiac functions in exposed mice, we adopted the following approach:

Let *X_ijkd_* be the average cardiac function measurement for mouse *j* in the *i*th group at a given period on the *d*th day of the *k*th week, where


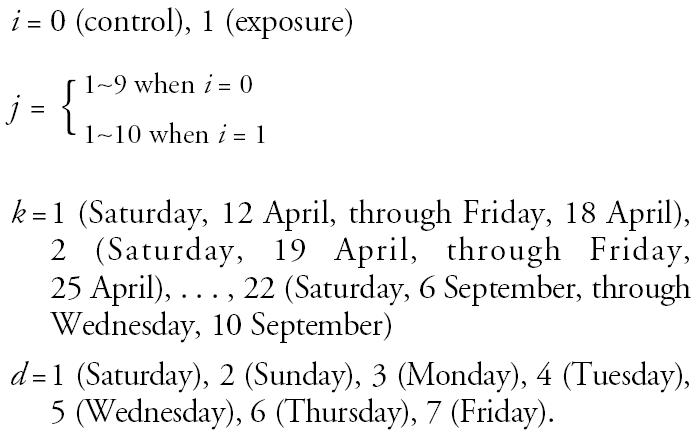


**Figure 1:**
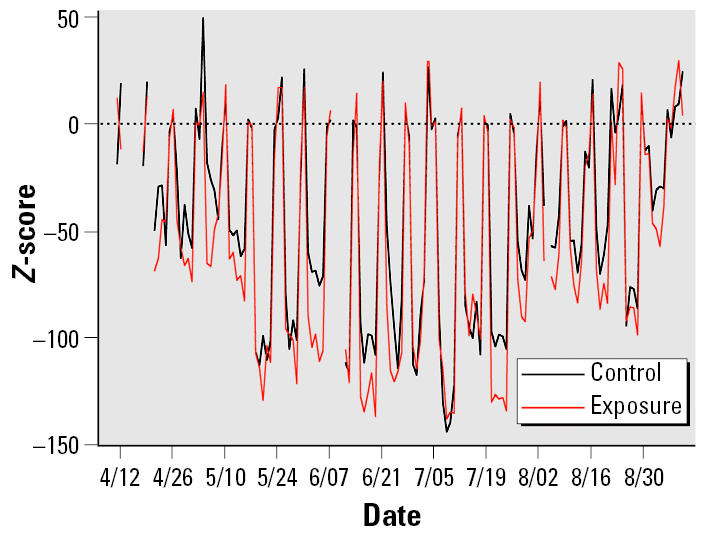
Daily average measurements of HR (bpm) for CAP-exposed and air sham–exposed (control) ApoE^−/−^ mice during the daily exposures (1100–1300 hr).

**Figure 2:**
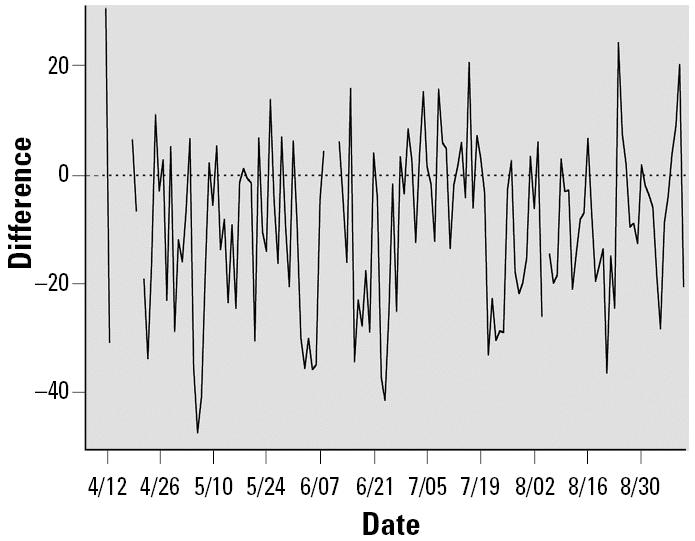
The difference (D) in HR (bpm) between CAP-exposed and air sham-exposed (control) ApoE^−/−^ mice during the daily exposures (1100–1300 hr).

We have seen that daily cardiac function measurements changed over the 5 months. Such changes may be caused by the cumulative effects of aging, exposure, and other unknown environmental factors. To examine the association between exposed level and acute cardiac function change on exposure days, we generated baseline adjusted measurements for each mouse on the exposure days by subtracting averaged measurement on the previous weekend from each measurement on weekdays. Presumably, the daily series of these baseline-adjusted measurements *Y_ijkd_* = *X_ijkd_* – (*X_ijk_*_1_ + *X_ijk_*_2_)/2 will have little cumulative effect. To see whether the idea worked or not, we explored the data. Figure 1 shows two series of daily averaged baseline adjusted measurements of HR at the 1100- to 1300-hr period for mice in the control and exposure groups. The exposure chamber effects reduced HR in both groups, which also corresponded to the quiescent period of mouse circadian rhythm during the daytime. The two series also share the same quadratic shape. Although it is not clear why this has happened, some common factors have strong effects on measurements of mice in both control and exposure groups. Instead of searching for a smooth curve for modeling the pattern caused by common factors, we can simply use the baseline-adjusted measurements of the nine mice in the control group to calculate an average for each exposure day. That is the darker curve plotted in Figure 1. If there is no exposure effect, the darker curve and lighter curve of averaged measurements for mice in the exposure group will not differ. In fact, the difference between two curves shown in Figure 2 indicates that CAP exposure had the effect of reducing HR. The difference series in the plot also show no trend over the 5 months, indicating that cumulative effects have been removed. Hence, we may construct a model to fit the baseline-adjusted measurements for examining whether the short-term cardiac function changes are related to exposure levels of the identified source factors F1, F2, F3, and F4. A linear model is given by


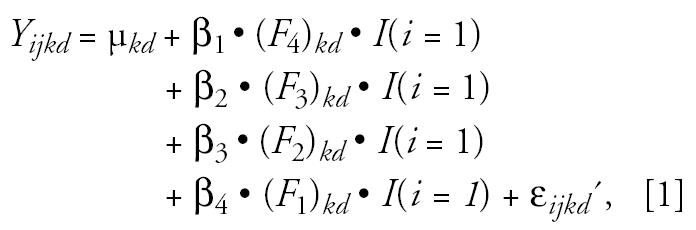


where ɛ *_ijkd_* is an autoregressive process of order one. If the estimate of β *_h_* differs significantly from zero, we may claim that the *h*th source factor is associated with the acute changes of HR and HRV.

## Results

### Associations between sources and short-term HR changes.

Using the source apportionment factors from Maciejczyk and Chen (2005), we have the following four source classes: SS, RS, RO, and MV. There were no significant associations between these four source categories and HR in the C57 normal mice at any of the three intervals. However, as shown in Table 1, there were highly significant associations between PM_2.5_ and the RS source factor and decreases in HR for the ApoE^−/−^ mice during the daily CAP exposures but no associations with the other source factors. By contrast, Tables 2 and 3 indicate that there was no residual association of HR with PM_2.5_ or the RS factor later in the afternoon or late that night.

In the afternoon, there was a significant association between decreases in HR and the SS factor for the ApoE^−/−^ mice that had not been present during exposure and did not persist into the nighttime period. It is also of some interest that the MV traffic and other source category was not significantly associated with HR during any of the three time periods.

For the C57 mice, there were no significant associations of HR with PM_2.5_ or any of its component source classes during any of the three daily time periods.

### Associations between sources and short-term HRV changes.

It is unfortunate that there was too much signal noise during the exposures to permit reliable analyses of HRV changes during the hours of CAP exposure. We therefore cannot tell whether the transient effect of PM_2.5_ or its RS source component on HR was also present for HRV. For C57 mice, the only significant association was between the MV and other source factor and a decline in RMSSD during the afternoons after the exposures (*p* = 0.00; data not shown). For the ApoE^−/−^ mice (Table 4), there were very strong associations of HRV with the RO source factor in the afternoon. These decreases in HRV did not persist at night (Table 5) and had not been seen for HR at any time period. Finally, there were strong associations between HRV during the nighttime hours and both the SS source category and the RS source category that were not seen for HR at the other intervals, or for HRV at the other time periods. However, it must be noted that although the SS source factor was associated with decreased HRV, the RS source category was associated with an increase in HRV. For PM_2.5_, there was a significant (*p* = 0.03) decrease in RMSSD and a nearly significant (*p* = 0.07) decrease in SDNN for the 0130- to 0430-hr interval but no such an association during the 1600- to 1800-hr period.

## Discussion

Interpretation of the various significant (*p* < 0.05) associations between source factors and the HR and HRV variables in CAP-exposed mice at this time would be speculative at best, especially because three of the source factors showed some association at one interval or another, and the fourth (MV traffic and other category) showed a strong association (*p* = 0.00) with RMSSD in the afternoon after exposure in the C57 mice. The strongest associations for the ApoE^−/−^ mice are summarized in Table 6.

For the evaluation of the changes on HR and HRV in the last column of Table 6, we have calculated the changes in the measured parameters over the interquartile range of concentrations as is commonly done in epidemiology. For HR, the changes are for exposures at the third quartile to the first quartile of the measured concentrations. The results show about 3–4 beats/min (bpm) changes. For HRV, the interquartile change is the ratio of RMSSDs between the third quartile and first quartile of the concentrations. The results show about 2–6% changes. These are relatively small changes, but they may have played some role in the progressive changes in HR that we observed during the course of the 5 months of exposure that were described by Hwang et al. (2005), and the changes in HRV that were reported by Chen and Hwang (2005).

It is also interesting that the reduction in HR during the daily exposures associated with PM_2.5_ (−4.1 bpm) may have been due entirely to the influence of the RS factor (−4.5 bpm) and that there was an increase in HR (+2.6 bpm) in the afternoons after the exposures in the same source factor. This appears to have been compensated by the decrease in HR in the afternoon after the exposures (−2.5 bpm) associated with the SS factor. Such a compensation would be consistent with the lack of any association of HR with PM_2.5_ in this interval.

The RO combustion factor, which did not have any significant association with HR, appears to have had the effect of increasing RMSSD by 6.2% during the afternoons after the exposure but not at the other intervals. The other observed statistically significant changes in RMSSD were associated with opposite effects during the late night period by the RS and SS source components, with the SS factor perhaps accounting for the significant association in the same direction for the association of RMSSD with PM_2.5_ during the same period.

It is also of interest that the effects reported here for HR and HRV were occurring at relatively low concentrations of outdoor PM_2.5_ and its component source-related factors. The average PM_2.5_ CAPs during the 6-hr exposures was only 113 μg/m^3^. Thus, the 24-hr average exposures were only 28.3 μg/m^3^ because the mice were breathing air that was filtered of the outdoor air components during the balance of the day. Outdoor PM_2.5_ does not have much diurnal variation, and it infiltrates indoors with a high degree of penetration. People are therefore exposed to concentrations of PM_2.5_ of ambient origin at near ambient concentrations for 24-hr each day. If indeed the ApoE^−/−^mouse is a good model for people with atherosclerosis, and if the HR and HRV responses to CAPs in these mice seen in this study are relevant to them, then such responses may be occurring in this human subpopulation at current ambient levels on many days each year.

There have been no previous reports that examined such responses at various periods during and after daily exposures. The only report of different ambient air PM source categories having different lagged effects was that of the PM Source Apportionment Workshop, in which human mortality effects were associated with different days of lag (Thurston et al. 2005).

Although there have been no previous reports of cardiac function effects that go in opposite directions after low-level environmental exposures, there have been such examples for other physiologic responses. In previous work in this laboratory, we reported in both humans (Leikauf et al. 1981) and rabbits (Schlesinger 1985) that short-term inhalation of a low concentration of submicrometer sulfuric acid aerosol increased the rate of mucociliary particle clearance from tracheobronchial airways, whereas a higher concentration (1 mg/m^3^) retarded such clearance. Similarly, the inhalation of the fresh smoke from two cigarettes accelerated tracheobronchial particle clearance in both humans and donkeys, whereas the smoke from 10 or more cigarettes slowed the particle clearance in donkeys (Lippmann et al. 1982). In another study in this laboratory, Schlesinger (1989) examined the effect of 14 days of sulfuric acid inhalation on particle clearance from the pulmonary region of rabbit lungs and found that low-level exposures accelerated such macrophage-mediated clearance, whereas higher levels of exposure retarded the clearance. In addition, subchronic inhalation exposures to both cigarette smoke and sulfuric acid produced persistent changes in particle clearance (Lippmann et al. 1987).

The fact that three different source factors showed some indication of a strong association with either HR or HRV in ApoE^−/−^ mice in this study, with the SS source component having an effect in the opposite direction to that of the RS source component, illustrates the complexity facing researchers when designing studies to identify the causal factors for the PM-associated adverse health effects reported in the epidemiologic literature. It may well be that most, if not all, PM source categories have some, if various, effects on cardiac physiology, with various lag structures, and that some components mitigate the effects produced by other components. Also, we do not know at this time about the short-term effects, and their temporality, of inhaled PM_2.5_ on other organ systems. However, if most of the major components of PM_2.5_ produce some short-term biologic responses, then the commonly used integral measure of PM_2.5_ mass concentration, that is, 24-hr average PM_2.5_, may be serving as a reasonable integrating index for at least some of the short-term health risks. In any case, the results reported in this article provide us and others with additional factors to consider in the planning of our future laboratory and field studies of PM health effects.

As noted above, Maciejczyk and Chen (2005) reported that *in vitro* NFκB expression of BEAS-2B cells exposed to CAPs collected during the daily 6 hr *in vivo* exposures was significantly increased in association with the RO, but not with the other source categories of the CAPs. The NFκB expression is an index of cellular oxidative stress and the release from the cells of mediators affecting systemic inflammation. This mechanism for biologic response is consistent with short lag times between respiratory tract particle deposition and cardiac function changes. However, because the NFκB index of biologic response to CAP exposure provides no within-day temporality, it is not possible to make a direct comparison with the lagged HR and HRV responses reported in this article. The different lag structures of the responses reported in this article may be related to the solubility of the biologically active components in each source category.

We plan to pursue the issues raised by the results reported here in our future subchronic exposure studies in mice. In terms of comparable investigations in humans, a study would need access to a population that is being continuously monitored for cardiac function as well as time-resolved PM_2.5_ compositional data. The only study we are aware of to date looking for cardiac responses to ambient air PM was by Sullivan et al. (2005), in which they examined the relation between PM_2.5_ exposure (measured by nephthelometry) and the number of hours preceding the onset of myocardial infarction (MI). They found no significant associations between MI and the nephthelometry data. It is possible that nephthelometry measurements may not be representative of the active components of ambient PM mix or that the nephthelometry measurements correlate with outcomes other than MI.

## Conclusions

The availability of data on HR and HRV over a 5-month period during subchronic exposures of mice to the regional anthropogenic CAPs at New York University’s Sterling Forest laboratory in Tuxedo, New York, and during the afternoon and nighttime periods after the daily exposures, as well as elemental composition data for each day’s exposure, enabled us to examine daily source apportionments of the major source categories during the exposures and their association with HR and HRV during each of the three time periods. The RS component was strongly associated with a transient decrease in HR during exposure, comparable with that of the whole PM_2.5_. The SS component was strongly associated with a transient HR decrease in the afternoon after the day’s exposure. The RO component was strongly associated with increases in HRV in the afternoon after the day’s exposure. The SS and RS components were strongly associated with HRV in the nighttime period, with decreased HRV for the SS component and increased HRV for the RS component. These effects were occurring after exposures at daily average PM_2.5_ concentrations occurring frequently in the United States and may be relevant to the subpopulation with atherosclerotic disease.

The biologic bases for these various associations and their temporal lags are not known at this time but may relate to the differential solubilities of the PM components at the respiratory epithelia and their access to cells that release mediators that reach the cardiovascular system. Further research that can elucidate the underlying processes is clearly needed.

## Figures and Tables

**Table 1:** HR parameter estimates for the 1100- to 1300-hr period for ApoE^−/−^ mice.

PM source component	Value	SE	*t*-Value	Pr(> |t|)
SS	−3.78E–02	2.41E–02	−1.55	0.12
RS	−3.61E–01	1.40E–01	−2.57	0.01
RO	−6.61E–01	7.23E–01	−0.91	0.36
MV	7.91E–02	2.44E–01	−0.32	0.75
PM_2.5_	−4.77E–02	1.33E–02	−3.59	0.00

**Table 2:** HR parameter estimates for the 1600- to 1800-hr period for ApoE^−/−^ mice.

PM source component	Value	SE	*t*-Value	Pr(> |t|)
SS	−3.63E–02	1.82E–02	−2.00	0.05
RS	2.09E–01	1.07E–01	1.96	0.05
RO	5.92E–01	5.79E–01	1.02	0.31
MV	2.36E–01	1.94E–01	1.22	0.22
PM_2.5_	6.55E–03	9.76E–03	0.67	0.50

**Table 3:** HR parameter estimates for the 0130- to 0430-hr period for ApoE^−/−^ mice.

PM source component	Value	SE	*t*-Value	Pr(> |t|)
SS	3.83E–02	2.02E–02	1.89	0.06
RS	−8.79E–02	1.18E–01	−0.74	0.46
RO	−4.12E–01	6.23E–01	−0.66	0.51
MV	−1.62E–01	2.08E–01	−0.78	0.44
PM_2.5_	−7.46E–03	1.10E–02	−0.68	0.50

**Table 4:** HRV parameter estimates for the 1600- to 1800-hr period for ApoE^−/−^ mice.

	Ln RMSSD (sec)				Ln SDNN (sec)			
PM source component	Value	SE	*t*-Value	Pr(> |t|)	Value	SE	*t*-Value	Pr(> |t|)
SS	3.74E–04	2.81E–04	1.33	0.18	−4.40E–06	2.77E–04	−0.02	0.99
RS	−2.20E–03	1.67E–03	−1.32	0.19	−1.90E–03	1.65E–03	−1.15	0.25
RO	2.64E–02	9.11E–03	2.89	0.00	2.62E–02	9.13E–03	2.87	0.00
MV	−3.57E–03	2.98E–03	−1.20	0.23	−4.41E–03	2.95E–03	−1.49	0.14
PM_2.5_	1.42E–04	1.51E–04	0.94	0.35	−1.18E–04	1.48E–04	−0.80	0.42

**Table 5:** HRV parameter estimates for the 0130- to 0430-hr period for ApoE^−/−^ mice.

	Ln RMSSD (sec)				Ln SDNN (sec)			
PM source component	Value	SE	*t-*Value	Pr(> |t|)	Value	SE	*t*-Value	Pr(> |t|)
SS	−1.07E–03	2.44E–04	−4.38	0.00	−9.28E–04	2.36E–04	−3.94	0.00
RS	3.40E–03	1.43E–03	2.38	0.02	2.43E–03	1.38E–03	1.76	0.08
RO	−3.92E–03	7.59E–03	−0.52	0.61	−1.44E–03	7.32E–03	−0.20	0.84
MV	3.29E–03	2.51E–03	1.31	0.19	3.22E–03	2.42E–03	1.33	0.18
PM_2.5_	−2.86E–04	1.32E–04	−2.16	0.03	−2.08E–04	1.28E–04	−1.63	0.10

**Table 6:** Short-term cardiac function changes associated with PM components with some significant *p*-values.

					Concentration (μg/m^3^)			
PM source component	Time of day (hr)	Affected variable	Effect coefficient (× 10^−3^)	*p*-Value	Mean	First quartile	Third quartile	Interquartile change
PM_2.5_	1100–1300	HR	−47.67	0.00	113.0	55.21	141.48	−4.1 bpm
RS	1100–1300	HR	361.23	0.01	13.18	5.88	18.36	−4.5 bpm
RS	1600–1800	HR	209.46	0.05	13.18	5.88	18.36	2.6 bpm
SS	1600–1800	HR	−36.30	0.05	63.41	25.08	79.20	−2.5 bpm
RO	1600–1800	RMSSD	26.37	0.00	1.53	0.01	2.30	6.2%
SS	130–430	RMSSD	−1.07	0.00	63.41	25.08	79.20	−5.6%
RS	130–430	RMSSD	3.40	0.02	13.18	5.88	18.36	4.3%
PM_2.5_	130–430	RMSSD	−0.29	0.03	113.0	55.21	141.48	−2.4%
